# An Apology for the Nerves; or Their Influence and Importance in Health and Disease

**Published:** 1845-07

**Authors:** 


### BIBLIOGRAPHICAL NOTICES.

*An Apology for the Nerves; or their influence and importance in health and disease.* By SIR GEORGE LEFEVRE, M. D., Fellow of the Royal College of Physicians, late Physician to the British Embassy at the Court of St. Petersburg, &c. &c. 12mo. pp. 363. London, 1844.

He, who sets out with a professed determination to prepare an apology for any thing, is apt to find his undertakiug terminate in an overstrained eulogy. Instead of acting as the unbiassed judge, he irresistibly argues as the interested advocate. Such, to a certain degree, is the character of Sir Geo. Lefevre's production. Thinking, that the nerves have been too much overlooked in pathological and therapeutical considerations, he has assigned to them influences which are often doubtful; and at times, we think, unfounded. For a number of years Sir George resided in St. Petersburg, and not long ago returned to his native country, soon after which he published his "*Life of a Travelling Physician*," which we perused at the time with pleasure,—not that it was characterized by any profundity of reflection, but it was written in a lively style, and exhibited that the author was a tolerable observer, and was withal a humorist; so that in his description of persons, places and events, there was a freshness, and at the same time a frankness, that could not but be taking. His present work is more medical, but it possesses the same praiseworthy qualities as the other. He who consults its pages for profound views will be disappointed: at times, indeed, he will find views with which he can by no means accord; and yet, ever and anon, an expression occurs, which will please him by its raciness and happy style. Moreover, the detailed results of Sir George's observations on climes, and amongst people, that are new to the reader, will convey to every one information for which he must feel thankful. The following remarks from the preface are characteristic:

"The blood holds a most important place amongst the vital organs, if it can usurp this title, but it does not hold the first place. To it the muscle owes its power, the nerve its tone; from it all the secretions are prepared; but it does nothing of itself; all depends upon its vitality, which it derives from the nerves. In the further consideration of this subject we shall endeavour to establish its full claims, physiologically and pathologically, not endowing it with properties, nor attributing to it intelligence, but giving it its due rank in its triple alliance with muscle and nerve. "The Travelling Physician" sought protection under the authority of a *sliding scale* for changes in opinions during twenty years of his life; and as he is well aware that a similar scale is applicable to every branch of science, he cannot but feel that a long exile may not have permitted him to keep pace with others in noting the all but daily discoveries that have been made in physiology. But if he has not learnt all that has been done at home, he may comfort himself with the idea that he will have less to unlearn. How much has proved ephemeral in this short space of time! Sir Charles Bell's respiratory nerves had a local habitation, and a name, in anatomical text books and manuals. They have been erased from subsequent editions of the same works. Poisons were proved, as far as experiments could prove anything, to be introduced into the system by means of the blood. The doctrine was set at naught by other experiments, still more conclusive than the *experimentum crucis* of Magendie. Dr. Addison and Mr. Morgan maintained that the nerves alone were the operating agents, and that too upon the undeniable test of experiment. Other physiologists again maintain, that there was fallacy in these proceedings,—the old story,—and the general opinion seems to be in favour of absorption into the blood to account for the effects of poisons on the system. What are we to think, then, of the nerves,—to hear once more of a nervous fluid, visible, ponderable, palpable, expressible from tubes, when the idea of this structure has been rejected, reviled, and proved fallacious for nearly a century. If those who think they have arrived at the top of the tree, find that they have again to descend to the bottom, the author will have less cause for reproaching the frost and snows of Russia, which, if they prevented him from climbing so rapidly as he might have wished, may, at least, have had the merit of lessening the height from which he might have had to fall." p. 10.

Yet some of the views embraced by the author, he can scarcely be accused of "climbing" after! They ought rather, we think, to have been sought for, buried in the soil at the foot of "the tree." Where, for example, does he find the following opinion?

"The nerves and muscles must form the blood, which can alone invigorate them. Hence, then, we come at once to the dependencies of the nerves, muscles and blood upon each other; and it is in vain to attempt to isolate them in their mutual influences. They are collaborators in all the functions of life, but they are not co-equals." p. 10.

What naturalist, again, believes in the gratuitous assertion of Dr. Macculloch, selected by the author as a motto to his work, and blazoned on his title page—that "*without a nervous system there is no animal*,—there can be none; without a circulatory one there are myriads." !

We shall not attempt to follow the author in his various desultory observations scattered through the volume before us, but shall indulge ourselves in some random remarks on those that especially strike our attention. In treating of the blood, we have the following:

"In speaking of the quantity of blood in the system, great discrepancies exist in the opinions of physiologists upon this subject,—a difference allowing of a range from eight to thirty pounds. Sir Astley Cooper estimated it an ounce per pound of solid. Now seventy ounces of blood have been taken from the arm, one-fourth of the whole vital fluid, without causing complete exhaustion, and the system has rallied again under such a loss, which, if the nervous energy be not too much impaired, will be in time replenished." p. 30. And he adds in a note—

"Dr. Parry estimated it at 20 lbs. *Note.* A woman died of hemorrhagy, losing 26 lbs. From a full blooded young woman, who was beheaded, 25 lbs. were collected.—*Wrisberg.*"

The subject of muscular motion—voluntary as well as involuntary—could not escape the notice of an apologist for the nerves; yet it is a difficult task to attempt to show that the nervous influence communicates to muscles the power of contraction. The more, indeed, we reflect upon the matter, the more are we compelled to adopt the Hallerian view of a *vis insita,* or innate power of contractility or irritability, on which the nervous influence acts as an excitant, and which it tends to exhaust like any other excitant. The singular phenomena, exhibited in the interesting experiments of Dr. F. G. Smith detailed in another part of this number, would seem, indeed, to prove, that the heart—an involuntary muscle—goes on contracting for hours by a *vis insita,* and that the nervous stimulus is not even needed to develope its contractions. It was, at one time, thought the height of absurdity to ascribe the action of that viscus to a "pulsific virtue" which it possessed; yet, in reality, we mean little or nothing more, when we affirm, that its contractions, as well as those of the intestinal canal, are owing to something inherent in its structure.

Of phrenology Sir George Lefevre does not say much. He cites—evidently with approbation—the remark of Mr. Travers, that "the phrenological system owes its existence to the countenance which it derives from a twilight of truth, though only sufficient to serve as a beacon to the absurdities with which it is enveloped;" yet he considers it a subject "of too much importance to be discussed in a cursory manner, or by the *non idonei.*" Lavater, he thinks, "is too much forgotten in the present day. His facial angle" [we thought it was Camper's] "cannot but persuade those who study it carefully, that they can read the man in his physiognomy." Yet who ever pretended to "*read the man*" by his facial angle!

The following is a merited tribute to a scientific and excellent individual—the discoverer of nitrogen—who was rapidly quitting the stage when we knew him. We well recollect the feelings entertained towards him as a practitioner by the mass of students, who could not comprehend his judicious treatment, but were on the look out—as has been the case amongst medical students in almost all time—for something heroic and startling, or at all events "decided."

"When in Edinburgh," says the author, "I was clinical clerk to the late Dr. Rutherford, with whose memory are associated most grateful recollections, and from whom I received most marked kindnesses. His practice was considered by the students, who know so much, or think they know so much, as puerile and inert, and his visits drew but few followers into the wards. Cases which seemed to us to demand the most active treatment and a free use of the lancet, were treated by a saline diaphoretic mixture, and a foot-bath, and got well under such treatment. He once said to me, with a smile, my practice differs from that of my colleagues; but it is my object to let the students see how much nature will do in many instances, and how patients recover under the most simple treatment, and by the removal only of all exciting causes. It is too common for you all to ascribe everything to the medicines you administer. The very active treatment you employ relieves the symptoms, and you take great credit to yourselves for your decided practice; but you forget that long convalescences follow, and the constitution is shattered and impaired by such abstraction of its powers. You will find that patients who have been apparently cured by large bleedings, which have conquered pain in the first instance, remain eventually longer in the wards than those who have not been so speedily relieved; moreover, you will find them return again, after their dismissal, with dropsy and chronic affections. As regarded bleedings, the Doctor never took away more than from eight to twelve ounces at a time; and now, after a period of twenty-five years his views of things, and those of his colleague, Dr. Gregory, seem to have been correct. For, as the latter has beautifully expressed it, "Nam uti sanguis semel missus nunquam in venas redit: sic nequè vires cum illo amissæ in variis morbis unquam refici possunt.

The change in this part of the treatment of disease has undergone great modifications, and practitioners no longer boast of their bleedings *ad deliquium*, which they did formerly. This *decided* practice, as it was styled, and which usurped the claims of confidence, is now proved to have been decidedly bad." p. 67.

The author adds two more cases, to the great number already recorded, of fatal results from the application of blisters in scarlatina. In one case tetanus supervened;—in the other gangrene.

Delirium tremens, he says, is a very common disease in St.

Petersburg; and, with great candour, he adds—" it always proved fatal in the cases which I treated. In the naval hospital, where it is very prevalent, opium and musk sometimes succeeded in saving the patient." p. 177. This uniform want of success is very unaccountable. It certainly is not a fatal disease here, when properly managed. We happen to have before us, at this very moment, an abstract of the number of patients admitted into the female Insane Department of the Philadelphia Hospital, from the first of November, 1844, to the 4th of February, 1845. In this quarter there were received, thirty-two cases of decided delirium tremens; and eighteen which are classed as intoxication. Of these not one died. The treatment was eclectic, often expectant, and not a dose of alcohol was given.

In the present work, as in his " *Thermal Comfort,*" Sir George touches upon some peculiarities of German Therapeutics, yet we see little worthy of having been recorded by him, and therefore little worth extracting by us.

The *grape cure, cure du raisin* or *Traubencur,* like the *Wassercur,* is one of those exclusive systems of treatment for which the Germans have been celebrated; and the efficacy of which reposes, by the way, in a great measure on this very exclusiveness. Revulsion appears to be the main agency, under the new impressions that are made by an entire revolution of diet and regimen: but let us hear what Sir George says of it.

" Those who have practised in Russia will have been made conversant with the cure du raisin. I had an opportunity of becoming so, when in the south of the empire, and in a grape country. It is necessary to state in what this cure consists, and for what class of diseases it is recommended. The latter may be dismissed at once, by stating, that all those functional nervous affections, which resist the routine of treatment generally employed, are the cases which may be so benefited, seeing that the discipline is more intolerable than the disorder for which it is instituted. A lady of rank leaves her bed of down and cushioned canopy, and migrates into the country,—turns a poor family out of their habitation,(not without making them an ample recompense,) and becomes the tenant of a filthy hut. This is part of the cure, viz.: to forego all luxury, to sleep in the peasant's crib, to sit upon his bench, and to avoid anything in the shape of comfort. The grape alone for meat, the grape for drink; a small quantity of dry bread is perhaps allowed. This is continued for the space of three weeks, and it is no wonder, if all circumstances are taken into consideration, that a cure is effected. I have known people of the highest rank subject themselves to such discipline, and have full faith in its results. It is homœopathy and hydropathy in another shape, and as the Italians say of all the varieties of form, in which they make their pastes, c'est toujours du macaroni." p. 282.

Then follow some observations, which sufficiently demonstrate that Sir George does not belong to the class of "teatotallers," as he terms them; and he dismisses the subject with the remark, that "they who have undergone the discipline of the grape-cure for a month are glad to come back again to the more comfortable liquor, which, when used, and not abused, is often one of the greatest blessings." p. 283.

But our space admonishes us that we must pause. In an appendix, the author has made some observations on different topics, which did not seem to him to be exactly adapted to the body of the work. On none of these do we think it necessary to dwell. One of them is properly headed "*credat judæus.*" It consists of an extract from the Collect. Academ. in the Romanzoff Library, St. Petersburg, and details the history of a woman who was confined of a female child, whose abdomen was larger than usual, and which child, eight days after birth, was itself delivered of a child of the length of the middle finger; "and as it was born," the chronicler adds, "and had the human figure, it was baptized, *mais la petite accouchée et sa petite fille moururent toutes les deux le lendemain.*" p. 324.

Sir George Lefevre obviously, from the caption of the article, does not believe this story, but by his publishing it without comment, there may be those who think that he reposes confidence in it. He, at all events, deems it worthy of being cited. It might have done for the pages of Pontoppidan, or for the article *Cas rares* in the *Dictionnaire des Sciences Médicales.*

